# A Novel Laser Energy Ablation Catheter for Endocardial Cavo-Tricuspid Isthmus Ablation and Epicardial Ventricular Lesion Formation: An *in vivo* Proof-of-Concept Study

**DOI:** 10.3389/fmedt.2022.834856

**Published:** 2022-03-21

**Authors:** Dennis Krist, Dominik Linz, Ulrich Schotten, Stef Zeemering, Dwayne Leenen

**Affiliations:** ^1^Cardiovascular Research Institute Maastricht (CARIM), Maastricht University, Maastricht, Netherlands; ^2^Department of Cardiology, Maastricht University Medical Centre +, Maastricht, Netherlands; ^3^Department of Cardiology, Radboud University Medical Centre, Nijmegen, Netherlands; ^4^Rheinisch-Westfälische Technische Hochschule (RWTH) Aachen University, Aachen, Germany

**Keywords:** cardiac arhythmias, laser catheter, ablation < electrophysiology, electrophysiology, linear ablation catheter, ventricular tachycardia

## Abstract

**Aim:**

This proof-of-concept study aimed to investigate atrial and ventricular lesion formation by a 20-mm linear laser ablation catheter, regarding lesion depth and tissue damage.

**Methods:**

In total, 6 female swines underwent standard femoral vein access to introduce a novel 20-mm linear laser ablation catheter in the right atrium to perform endocardial cavotricuspid isthmus (CTI) ablations. The navigation took place under fluoroscopy with additional visualization by intracardiac echocardiograph. *Via* a sternotomy, epicardial ablations were performed on the surface of the left ventricle (LV), right ventricle (RV), and right atrial appendage (RAA). Procedural safety was assessed by registration of intraprocedural adverse events and by macroscopic examination of the excised hearts for the presence of charring or tissue disruption at the lesion site.

**Results:**

Altogether 39 lesions were created, including 8 endocardial CTI (mean lesion length 20.6 ± 1.65 mm), 26 epicardial ventricle (mean lesion length LV: 25.3 ± 1.35 mm, RV: 24.9 ± 2.40 mm), and 5 epicardial appendage ablations (mean lesion length RAA: 26.0 ± 3.16 mm). Transmurality was achieved in all CTI and atrial appendage ablations, in 62% of the RV ablations and in none of the LV ablations. No perforation or steam pop occurred, and no animal died during the procedure.

**Conclusion:**

In this porcine study, the 20-mm linear laser ablation catheter has shown excellent results for endocardial cavotricuspid isthmus ablation, and it resulted in acceptable lesion depth during atrial and ventricular epicardial ablation. The absence of tissue charring, steam pops, or microbubbles under the experimental conditions suggests a high degree of procedural safety.

## Introduction

Typical right atrial flutter is a common abnormal heart rhythm that frequently occurs in patients with atrial fibrillation. For the minimal invasive treatment of atrial flutter, a linear conduction block is created at the cavotricuspid isthmus (CTI). Ablation of the isthmus prevents conduction at the narrowest point of the circuit and therewith usually terminates atrial flutter as the block is being completed. The use of a linear ablation catheter could improve procedure times and minimize conduction gaps.

In the area of epicardial procedures, linear ablations could also be beneficial in the treatment of ventricular tachycardias (VT) or persistent atrial fibrillation (1). Currently, no uniform agreement exists on the optimal VT ablation strategy. However, high-density delineation of the scar is commonly the first step (2). Various ablation strategies can be applied to reduce scar-related recurrent VT. Scar homogenization is an important trend to eliminate all abnormal electrograms by extensive and diffuse ablation at the scar (3, 4). More extensive and linear ablations of large scar-related ventricular tachycardia are associated with a better success rate and a lower recurrence rate (5). Biophysical limitations of RF ablation are constituted by the thick ventricular walls with transmural circuits, where, besides the wall thickness, trabeculations and fat act as barriers to effective power delivery into a scar. A laser could be an improved technique to create deep and linear lesions. Besides, the unidirectional emission of laser light does not damage any surrounding tissue such as the phrenic nerve, the lungs, and the parietal pericardium. Therewith, deep linear lesions would allow epicardial substrate modification in the setting of postinfarct scar reentry VT, but could also provide an additional method for epicardial left atrial ablations in the treatment of longstanding persistent atrial fibrillation (6, 7).

This proof-of-concept study was designed to investigate the performance of a novel deflectable 20-mm linear laser ablation catheter and to create transmural endocardial CTI lesions and epicardial atrial and ventricular lesions with a focus on lesion depth, tissue damage, and general procedural safety in a porcine experimental model. Additionally, this data was used to validate the *ex vivo* models of the preclinical dose-finding studies.

## Materials and Methods

### Animal Preparation

The experiments were performed at the Cardiac Physiology Experimental Lab of the Charles University and Na Homolce Hospital (Albertov 5, 128 00 Prague, Czech Republic). The experimental protocol was compliant with the local guidelines regarding animal experiments. Female swines with a weight between 40 and 60 kg and ~4–6 months of age were used for the experiments. Animals were inspected by a veterinarian for health status and exclusion criteria, fasted 12 h prior to the procedure with unlimited access to water. An acute non-survival protocol was applied, with euthanization prior to recovering from anesthesia. Preanesthesia was initiated by intramuscular injection of ketamine and midazolam. After the effect, venous access was secured. In the operating room, the animal was placed in a supine position on the x-ray table and the ECG was attached. Total intravenous anesthesia (propofol, midazolam, and morphine) was induced and continued throughout the procedure to maintain the surgical level of anesthesia. Orotracheal intubation was performed and mechanical ventilation was initiated. Anticoagulation was maintained by Heparin to reach a target ACT of 350 s, which was checked hourly. Antibiotic bolus was applied when the expected experiment duration exceeded 4 h. Body temperature was controlled by heating or cooling the mattress to maintain 38°C. Standard femoral vein access was used to introduce a steerable sheath in the RA and to insert an ICE catheter (AcuNav 10F Ultrasound catheter of Siemens Healthineers AG, Erlangen, Germany) in the upper part of the RA. *Via* the jugular vein, a CS catheter was positioned. After the endocardial atrial ablations, a sternotomy was created and the pericardial sac was opened to gain access to the epicardial surface of the LV, RV, and RAA. After euthanization, the hearts were excised for macroscopy.

### Laser Ablation Catheter

The experimental 20 mm linear laser catheter was designed by Vimecon GmbH (Herzogenrath, Germany) (Patents: US 2018/0021089, US 2011/0230941, US 2011/0230871). It possessed a bidirectional steerable 8.2F shaft with a deflection angle of ~180° in both directions. The 20-mm laser active area was shaped slightly convex to optimize wall contact. A saline irrigated tip was used to dissipate the remaining energy in the optical fiber. Typical energy densities during the experiments were between 75 J/mm (for CTI ablation) and 150 J/mm (for LV ablation), as defined per *a priori ex vivo* dose-finding study.

**Figure 1:**
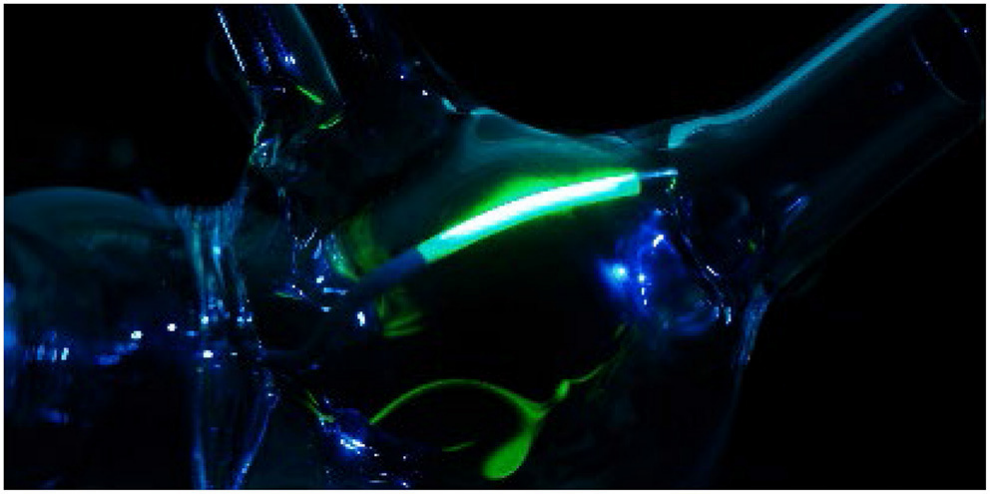
Visualization of the laser active area by use of a low energy green laser diode, depicting the linear lateral emission of the catheter.

Using a low-energy green laser diode, the laser active area was visualized, as depicted in Figure 1, demonstrating the lateral emission of the catheter. This design emitted laser light with a divergence of ~85°, with its highest intensity over the longitudinal axis of the catheter. Due to the large divergence, wall contact is indispensable to achieve well-formed lesions. During a successful ablation, the laser light is absorbed by the tissue, leading to thermally induced coagulation of the targeted substrate.

### A *Priori ex vivo* Dose-Finding

Through an *ex vivo* dose-finding study, the power and time settings for transmural lesions were defined. Avian tissue and freshly excised wporcine hearts with a thickness >10 mm were used as the substitute tissue model to estimate the energy-dependent lesion depth. The tissues samples were heated and kept at a temperature of 37°C ± 2°C in a saline solution bath. The lesion depth could be assessed macroscopically by a section at the target site due to the strong and clear border demarcation of the coagulated area. The power settings were varied between 20 and 30 W, and the time was set between 60 and 150 s. In total, 138 energy applications were performed on avian tissue and 38 applications on porcine cardiac tissue. By means of this animal study investigation, the *ex vivo* models were validated for their suitability for lesion predictability investigations.

### Statistical Analysis

For comparison of data, the following tests were used with a significance cut-off set at 5%: Pearson's correlation coefficient was determined to investigate the relationship between applied energy and lesion depth, and the Kruskal-Wallis test was used to assess the *ex vivo* and *in vivo* tissue models. For the statistical analysis, invalid *in vivo* measurements were omitted if strong ventricular contractions caused substantial movement of the catheter during the ablation procedure.

### Procedure Details

**Figure 2:**
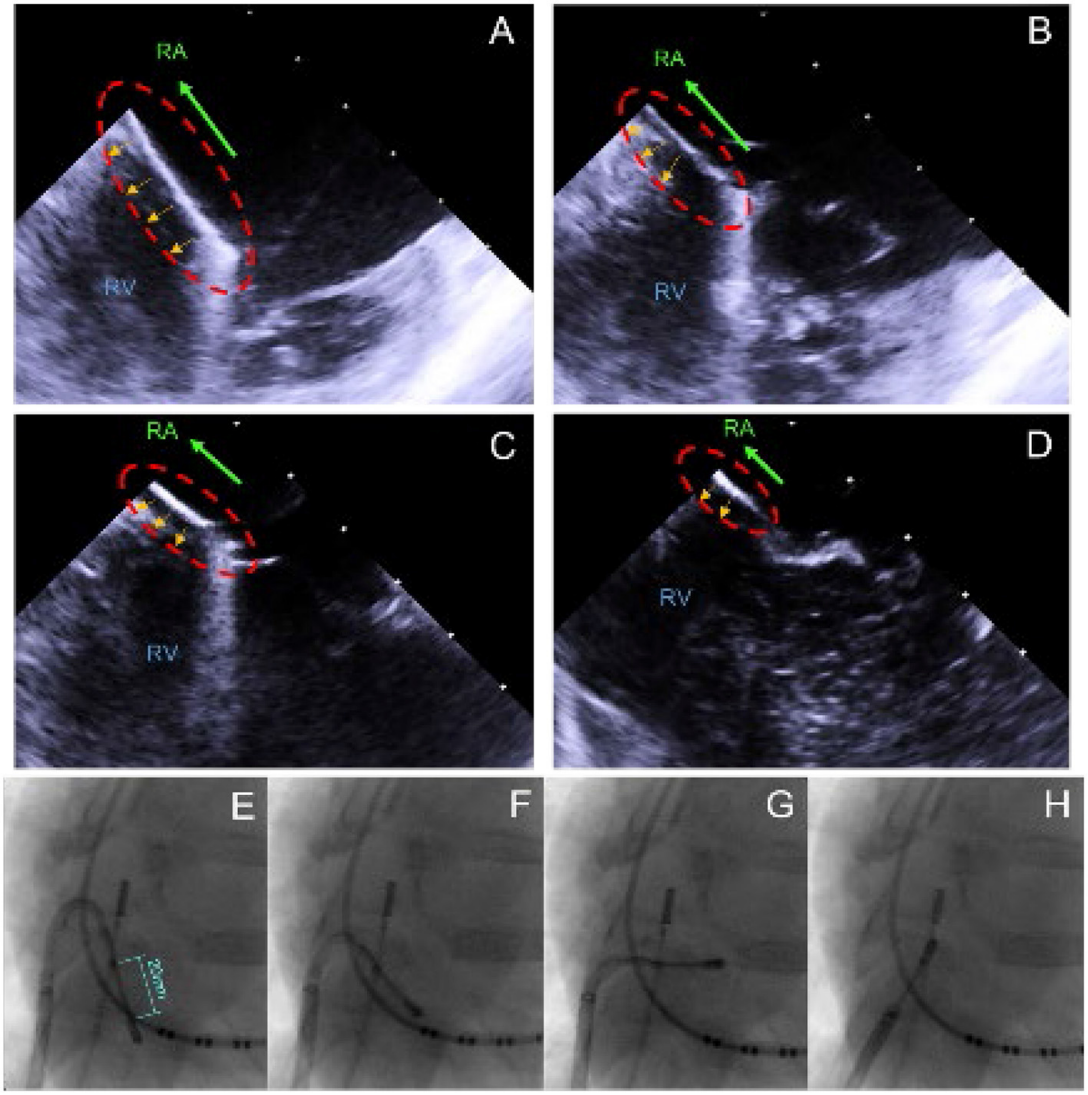
ICE/X-ray recordings captured during the CTI ablation, visualizing the step-wise retraction from the RV/TCV toward the IVC. **(A)** Initial position with the catheter partially in the RV and on the TCV. **(B–D)** Stepwise retraction into the IVC. **(E)** Initial position with the catheter partially in the RV and on the TCV. **(F–H)** Stepwise retraction into the RA.

In total, 6 female animals were prepared for the experimental ablations with the linear laser catheter. First, the endocardial ablations were performed by transfemoral access to the RA. The use of a 82 cm, 8.5F steerable sheath (Agilis Nxt, Dual-Reach, St. Jude Medical, Saint Paul, United States of America) enabled free movement in the RA and combined with the steerability of the catheter, it allowed good positioning of the catheter. The navigation took place under fluoroscopy and by manual control of the experienced physicians. ICE was used as the additional visualization tool. Figure 2 displays the recorded ICE and fluoroscopy images during navigation. To perform CTI ablation, the catheter was inserted partially into the RV over the tricuspid valve (TCV), as displayed in image A and E. It was followed by step-wise retraction toward the inferior vena cava (IVC) to create a linear lesion, extending from inside the TCV to the IVC, as depicted in images B-D and F-H.

The slight convex shape of the laser active area allowed good wall-contact over the entire length, adjustable *via* the bidirectional steering mechanism. The power and time settings were defined at 25 W/60 s, as per dose-finding. During the power application, the catheter was maintained at the same location as much as possible to assure transmurality.

The epicardial ablations of the RAA, RV, and LV were conducted under direct visualization *via* a full sternotomy, allowing free access to the atria and ventricles. The lesions were created with an adequate distance between the applications to prevent lesion overlapping in order to assess the length and depth per individual power application. The left ventricle ablations were deployed on the anterior wall between the left anterior descending artery and the circumflex artery. In the right, ventricle ablations were deployed between the left anterior descending artery and the coronary artery. Sites with pronounced epicardial fat or marginal and diagonal arteries were avoided.

### Procedural Safety

In addition to the endo- and epicardial lesion formation process, the procedural safety was assessed by awareness and recording of steam pops, acute procedural complications with respect to the vascular access site, severe arrhythmias, and pericardial effusion/tamponade. During power application, the target site was inspected for signs of microbubbles and clotting by use of ICE. Post-procedural inspection of the catheter, lesion site, and surrounding tissues was conducted to confirm the absence of charring and damage to surrounding tissue structures.

## Results

### Endocardial CTI Ablation

**Figure 3:**
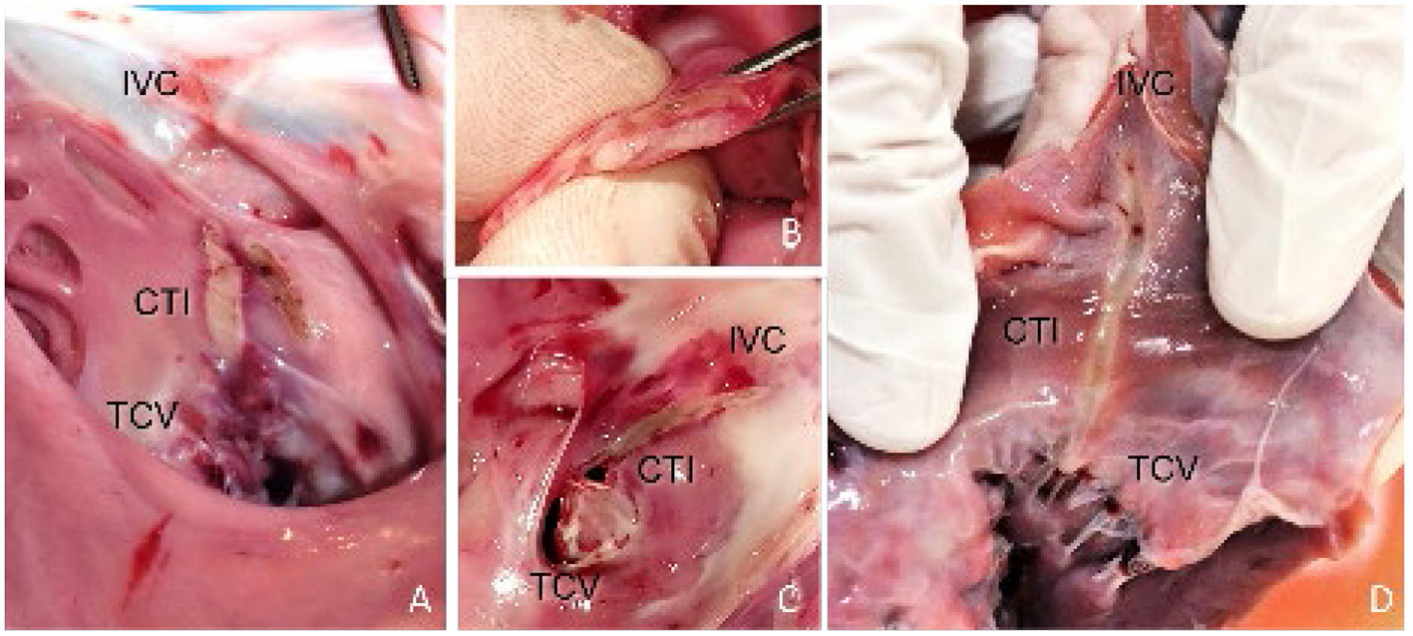
Endocardial view of the RA specimens displaying the pale linear lesion. **(A)** Incomplete CTI ablation, showing the created lesions of 4 power applications at 25 W for 60 s. One application was set parallel to the rest. **(B,C)** Full CTI ablation including axial section, confirming transmurality and linearity. The lesion extended from the TCV toward the IVC. **(D)** Full CTI ablation, requiring only two energy applications of 25 W/60 s to complete the lesion.

In three of the six animals, CTI ablations were attempted with the 20-mm linear ablation catheter. Good wall contact with sufficient force was consistently obtained by manual control using a steerable sheath, steerable catheter, and proper catheter orientation. Confirmation of wall contact was achieved *via* x-ray and visualized by the movement of the cardiac wall during catheter handling. ICE proved to be a useful tool to assess the exact location of the catheter and as an additional verification of wall contact. All ablations (*n* = 8) were transmural, with a mean lesion length of 20.6 ± 1.65 mm. A full CTI lesion from the TCV up to the IVC was obtained in two of the three animals. In the third animal, the lesions did not connect between the IVC and TCV due to gaps in the ablation line. In this procedure, the catheter was not retracted toward the IVC but repositioned by catheter navigation between applications. The CTI ablation was well-tolerated, and no sustained arrhythmias were induced during energy application. Figure 3 shows the macroscopic analysis of the CTI ablations in the excised hearts. In none of the performed ablations charring of the tissue surface, steam-pops or tissue disruptions were observed at the lesion site. Intraprocedural visualization of the ablation site with ICE also showed no signs of microbubbles during power application. On the catheter, no signs of charring or coagulation were found. Macroscopic analysis of the surrounding tissue structures showed no signs of laser ablation-related tissue damage. Autopsy of the lungs also showed no signs of emboli.

### Epicardial Ablations

**Figure 4:**
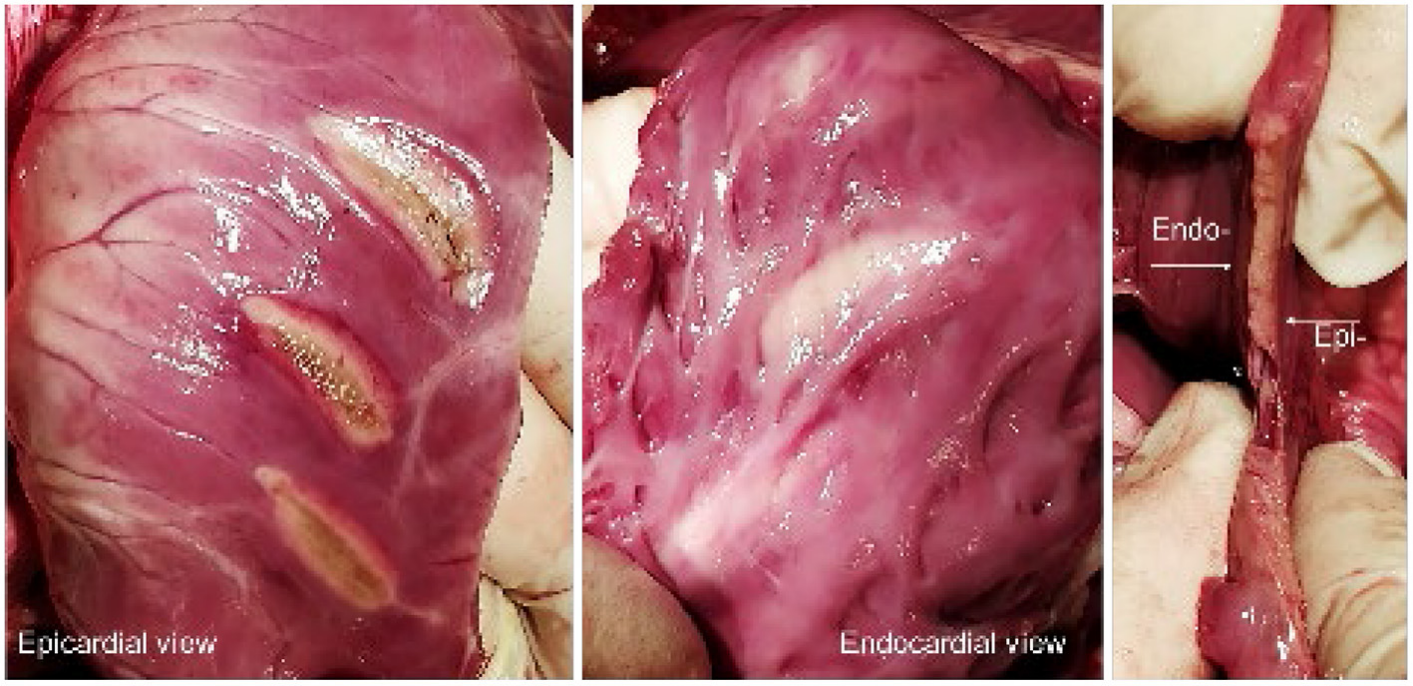
**(Left, Middle)** Epi- and endocardial view of three epicardial RV ablations. **(Right)** Axial section of a lesion, displaying the transmurality and linearity.

In all the 6 animals, epicardial ablations were performed on the ventricles and on the right atrial appendage. This resulted in 13 left ventricles, 13 right ventricle, and 5 appendage ablations, with a mean lesion length of 25.3 ± 1.35 mm (LV), 24.9 ± 2.40 mm (RV), and 26.0 ± 3.16 mm (RAA). Power and time settings were varied between 15 and 30 W and 60 and 180 s, respectively. Comparable with the *ex vivo* dose-finding, the time settings had more impact on the achieved lesion depth than power. It was observed that the maximum lesion depth, estimated at 9 mm, was achieved after 120 s regardless of whether 25 or 30 W of energy was applied. The findings correlate with the gathered *ex vivo* data, also indicating a maximum lesion depth of ~9 mm. In most RV ablations, transmurality was easily observed by clearly visible discoloration of the endocardial tissue surface, as depicted in Figure 4.

**Figure 5:**
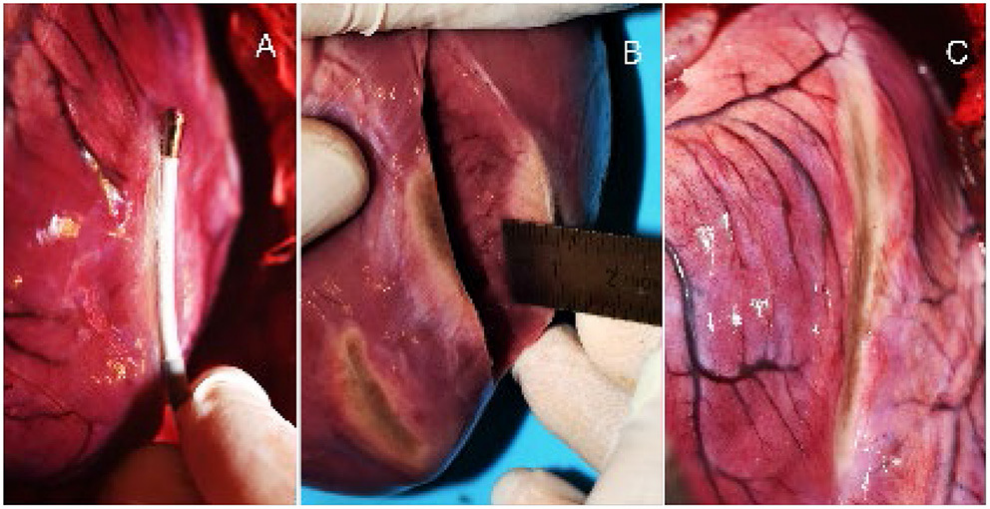
**(A)** Epicardial ablation of the LV with the laser ablations catheter. **(B)** Axial section of the excised heart and macroscopic estimation of the lesion depth. **(C)** 4 connected ablations with approx. length of 80 mm.

The presence of endocardial trabeculations mostly prevented full lesion transmurality, due to the increased tissue thickness and additional convective cooling. In general, transmurality was not achieved if the wall thickness surpassed 10 mm. Subsequently, transmurality could not be obtained in left ventricular ablations. All lesions demonstrated equal homogeneity and linearity over the length of the laser-active area. Dissection showed no signs of intramural hematoma. The macroscopic analysis of the LV ablations (Panel B) and the intraprocedural catheter positioning (Panel A) are depicted in Figure 5. Under the experimental conditions none of the performed ablations showed evidence of charring on the tissue surface, steam-pops, or tissue disruptions at the lesion site, suggesting a high degree of procedural safety. Epicardial bleeding was not observed. Non-sustained VTs and ventricular premature contractions were frequently induced from the second ventricle ablation or onward with spontaneous termination in a vast majority of cases.

### *In vivo* Validation of the *a Priori ex vivo* Dose-Finding Studies

**Figure 6:**
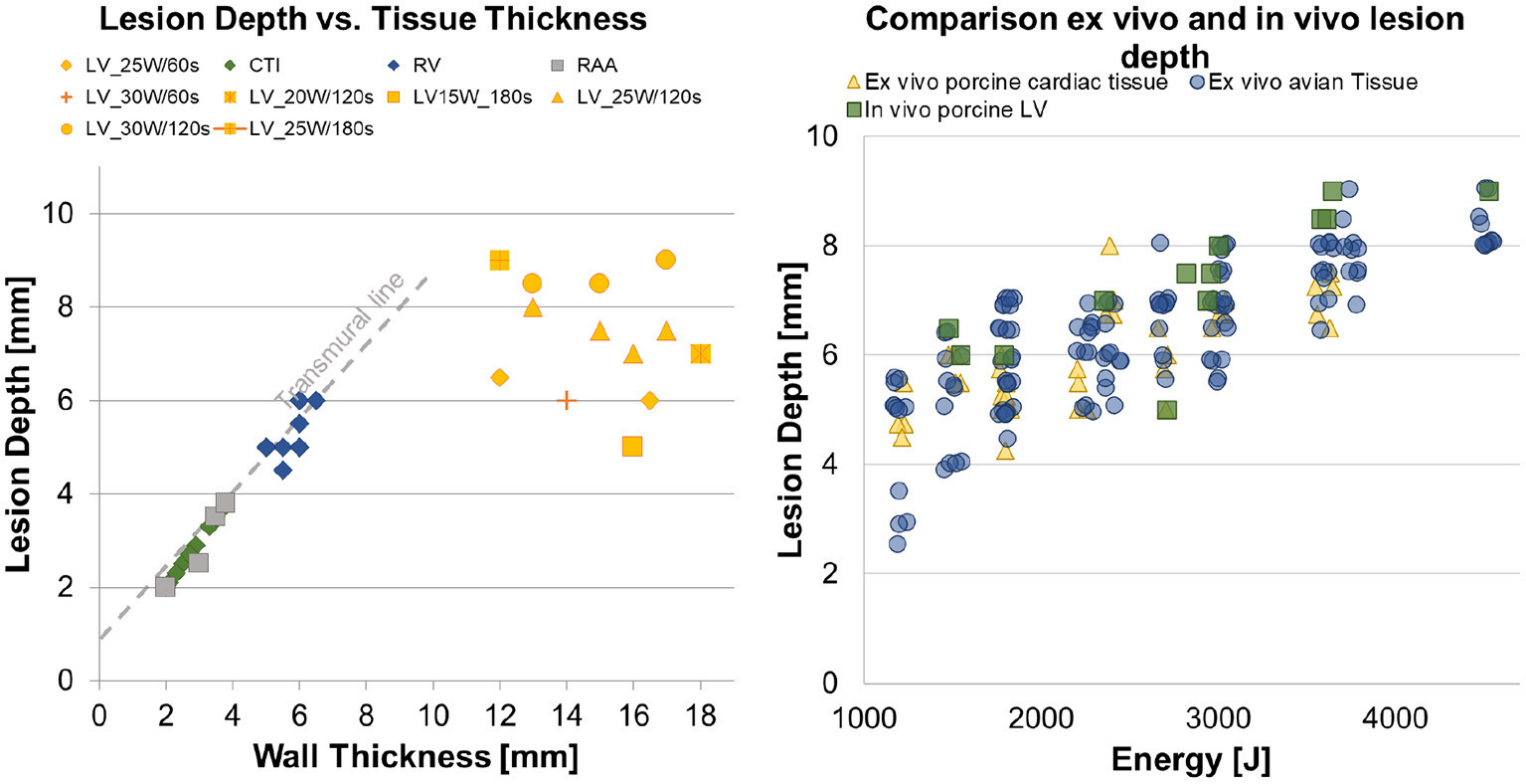
**(Left)** Overview of the performed CTI and epicardial ventricle ablations, with a differentiation between transmural and non-transmural ablations. **(Right)** Three scatter plots, representing the *in vitro* avian tissue model, the *in vitro* porcine cardiac tissue model and the results from the *in vivo* study.

In Figure 6 (Left Panel) all 39 lesions from this animal investigation were plotted against the estimated wall thickness of the targeted substrate. A transmural line was added to visualize the transmurality per lesion. For epicardial LV ablations, the association between wall thickness, applied power, and application time was displayed individually.

In the right panel, a scatter plot regarding energy-dependent lesion depth was created to represent the generated data from this *in vivo* investigation and the antecedently conducted *ex vivo* dose-finding studies. Pearson's product–moment correlation was applied to assess the relationship between applied energy and lesion depth for all groups. A strong positive correlation between the applied energy and the lesion depth was indicated, with a Pearson correlation factor of *r* = 0.706, *p* < 0.001 for the *ex vivo* porcine tissue, *r* = 0.798, *p* < 0.001 for the *ex vivo* avian tissue, and *r* = 0.659, *p* = 0.027 for the *in vivo* left ventricles. Table 1 lists the results for the Kruskal–Wallis test comparing the *ex vivo* and *in vivo* models regarding energy-dependent lesion depth. No significant difference in energy-dependent lesion depth was indicated between the *in vivo* and *ex vivo* studies.

**Table 1:** Statistical analysis.

**Tissue sample**	***N***	**Median**	**Median rank**	***H*-value**	***p*-value**
**Kruskal–Wallis test**
*Ex vivo* porcine	6	6.306	8.2		
*In vivo* LV	6	6.750	11.7	*H* = 1.51	*p* = 0.470
*Ex vivo* avian	6	6.438	8.7		

*A Kruskal–Wallis test was conducted to compare the energy dependent lesion depth of this in vivo animal study and antecedently conducted ex vivo dose-finding studies on porcine and avian tissue. No significant difference was indicated, with p > 0.05*.

## Discussion

In this proof of concept study, we sought to investigate the capability to create transmural lesions during CTI ablation and epicardial atrial and ventricular power applications with a deflectable 20-mm linear laser ablation catheter. To this aim, an *in vivo* animal study was conducted, which also considered procedural safety. The laser catheter showed to be effective in creating well-demarcated transmural endocardial CTI lesions without negatively affecting the procedural safety. Although all atrial lesions were transmural, this translates not directly into procedural success, since the main objective of atrial flutter ablation remains conduction block at the isthmus. In a setting without 3D electroanatomical activation and voltage mapping systems, this confirmation of lesion contiguity proved to be difficult. In future studies, the use of these systems is inevitable to prove the effectiveness of this laser catheter. At the same time, the direction of the lateral laser emission could be visualized. Since the laser ablation catheter already possesses an optical fiber, spectroscopy could be used to directly visualize the tissue and lesion formation. This principle was already proven to be feasible in several studies combining radiofrequency ablation and optical spectroscopy (8–10), allowing the detection of wall contact, prior lesions, and tissue defects.

Navigation was no issue during the epicardial ablations performed under direct visualization of the catheter and the target site, as a result of the midline sternotomy. The epicardial atrial and ventricle ablations created linear lesions without evidence of charring, steam-pops, or tissue disruptions. In the right ventricle, transmurality was obtained in more than half of the ablations. In the left ventricle, this could not be achieved due to the high wall thickness of up to 18 mm.

In comparison to previous epicardial RF ablation studies (11, 12), the laser ablation resulted in deeper and more homogeneous lesions. *Ex vivo* studies already showed that increasing the power above 30 W did not create deeper lesions. To reach lesion depths beyond 9 mm, a different or additional wavelength could be used. Past investigations have shown that 800 nm has at least 50% more penetration depth in comparison to 980 nm, providing an alternative to achieve transmural lesions in the left ventricle (13–15). Increased penetration depths have also been achieved by the use of pulsed wave laser light, leading to reduced energy absorption in the superficial layers of the myocardium (15). A different wavelength and pulsed-laser applications are easily implementable in the current catheter design since the same fiber optic components can be used.

An interesting observation was made comparing the lesion length of endo- and epicardial ablations. This showed a reduced length of ~5 mm for the endocardial ablations, which can be explained by the convex shape of the applicator and the convective cooling of the outer edges of the target site. The circulating blood in the right atrium cools the tissue around the catheter. Therewith, only directly irradiated areas are heated. The scattered energy beyond the area of direct irradiation is dissipated. Without the effects of convective cooling, the lesion length increases as observed in the epicardial ablations.

The macroscopic analysis of the excised hearts showed no edema formation after endo- and epicardial laser ablation. This could benefit the long-term freedom of AFL and procedural efficacy. Reversible acute conduction block induced by edema formation, which is indistinguishable from complete tissue necrosis, can be prevented (16, 17). This way conduction gaps formed with the passing of time are obviated. In future studies, this potential lack of edema-induced false conduction block needs to be investigated and observed more thoroughly.

Overall, the deflectable 20-mm linear laser catheter showed good performance regarding lesion depth and procedural safety in endo- and epicardial ablations. The achieved maximum ventricular lesion depths are slightly deeper than the conventional RF- and cryo devices (11, 12), but also compared to upcoming technologies like pulsed-field ablation (18). As for all thermal ablation devices, achieving tissue contact is critical for lesion formation when delivering energy. The linearity of the lesion could be beneficial toward RF power applications, reducing the needed amount of power applications and therewith reducing procedure times. In epicardial ablations, the unilateral laser emission holds a benefit in comparison to RF- and cryo applications since only the targeted tissue will be treated, protecting the surrounding tissue.

Besides the ablation of the cavotricuspid isthmus in patients with typical right atrial flutter, the mitral isthmus (MI) could be targeted as commonly applied in patients with atypical left atrial flutter (1). Although the linear catheter showed promising results in epicardial lesion formation and safety of laser application, the design needs further development to support substrate modification procedures where linear lesions are not required.

In closing, the achieved lesion depths of the LV ablations were used to validate the applied *ex vivo* tissue models regarding lesion depth, as used in preclinical dose-finding studies. No significant difference in lesion depth was observed between *in vivo* and *ex vivo* lesions. Therefore, the used *ex vivo* models are a valid substitute to predict the lesion depth and therewith to define the therapeutic ablation settings to achieve transmurality in the case of laser applications. The benefits of laser-based catheter ablation extend beyond the lesion formation process. The laser additionally allows online monitoring of the laser effects on the myocardium. This immediate and real-time verification of ablation success would be extremely beneficial to increase procedural success.

### Limitations

The ablations with the 20 mm linear catheter were always conducted as part of an animal trial study of the endocardial laser lasso catheter. Therefore, no ablations were performed in (endocardial) and on (epicardial) the LA to prevent any contamination of the created lesion with the lasso catheter. Accordingly, only the RV, RA, RAA, and LV were targeted during the combined endo- and epicardial ablations.

The used swine model showed a high amount of similarity with human anatomy and physiology, allowing extrapolation of lesion formation and procedural safety data. However, one aspect that could not be accounted for in this animal study is the usually challenging isthmus anatomy in humans (19). Common anatomical peculiarities like prominent Eustachian ridge, pouchlike recesses, and pectinate muscles are not observed in young swines where the surface is usually smooth and without trabeculations. The effect of anatomical peculiarities on the lesion transmurality needs to be assessed in a different model that reflects the human anatomy and all its characteristics. In these cases a more flexible catheter design could be better suitable for difficult anatomies or by adding tip emission, enabling point-by-point ablation in pouches.

As swines do not tolerate ventricle ablations very well, only a few ventricle ablations could be performed *in vivo*, i.e., with perfused tissue. The gathered data regarding epicardial lesion formation in the ventricles provide a good basis for further investigations. The endo- and epicardial atrial ablations on the other hand were well-tolerated. It needs to be mentioned that due to the small sample size of the *in vivo* ablations, more research is required to further substantiate the results of the study.

Moreover, the epicardial ablations were performed in an ideal setting, i.e., with an opened thorax. Thus, good contact was always achieved under visual navigation. Ablations were solely performed on healthy ventricular tissue, and therewith the effect on ventricular scar tissue could not be assessed. Though the effect can be estimated by studies conducted in the past on scar tissue, in future studies, these aspects could be investigated more specifically.

## Conclusion

The 20-mm linear laser ablation catheter has shown excellent results for endocardial cavotricuspid isthmus ablation and it resulted in acceptable lesion depth during atrial and ventricular epicardial ablation. There was no evidence of tissue charring, steam pops, or microbubbles under the experimental conditions, suggesting a high degree of procedural safety. Laser, as an energy source, for catheter-based epicardial ablation is promising but requires technical optimization of catheter design. Further studies are needed to assess long-term efficacy and safety.

## Data Availability Statement

The raw data supporting the conclusions of this article will be made available by the authors, without undue reservation.

## Ethics Statement

The animal study was reviewed and approved by Central Commission for Animal Welfare (Czech Republic).

## Author Contributions

DK contributed to conception and design of the study and wrote the first draft of the manuscript. SZ and DLi contributed to manuscript revision, read, and approved the submitted version. DLe contributed to execution of study and to manuscript revision. All authors contributed to the article and approved the submitted version.

## Conflict of Interest

The authors declare that this study received funding from Vimecon GmbH. The funder had the following involvement with the study: sponsoring the project, owner of intellectual property rights.

## Publisher's Note

All claims expressed in this article are solely those of the authors and do not necessarily represent those of their affiliated organizations, or those of the publisher, the editors and the reviewers. Any product that may be evaluated in this article, or claim that may be made by its manufacturer, is not guaranteed or endorsed by the publisher.
